# Irbesartan for the treatment of hypertension in patients with the metabolic syndrome: A sub analysis of the *Treat to Target *post authorization survey. Prospective observational, two armed study in 14,200 patients

**DOI:** 10.1186/1475-2840-6-12

**Published:** 2007-04-03

**Authors:** Ulrich Kintscher, Peter Bramlage, W Dieter Paar, Martin Thoenes, Thomas Unger

**Affiliations:** 1 Center for Cardiovascular Research (CCR), Institute of Pharmacology, Charité, Berlin, Germany; 2 Institute for Clinical Pharmacology, Medical Faculty Carl-Gustav Carus, Technical University Dresden, Germany; 3 Sanofi-Aventis Deutschland GmbH, Medical Affairs CardioVascularThrombosis, Berlin, Germany; 4 Sanofi-Aventis, Medical Affairs, Paris, France

## Abstract

**Objectives:**

The metabolic syndrome is a cluster of cardiovascular risk factors leading to an increased risk for the subsequent development of diabetes and cardiovascular morbidity and mortality. Blocking the renin-angiotensin system has been shown to prevent cardiovascular disease and delay the onset of diabetes. Irbesartan is an angiotensin receptor blocker (ARB) which has been shown to possess peroxisome proliferator-activated receptor gamma (PPARγ) activating properties, and to have a favorable metabolic profile. Current discussion is whether the addition of small doses of hydrochlorothiazide changes this profile. Therefore the efficacy, safety and metabolic profile of Irbesartan either as monotherapy or in combination therapy was assessed in patients with the metabolic syndrome in a large observational cohort in primary care.

**Research design and methods:**

Multicenter, prospective, two-armed, post authorization study over 9 months in 14,200 patients with uncontrolled hypertension with and without the metabolic syndrome (doctors' diagnosis based on the Adult Treatment Panel III criteria 2001). Blood pressure was measured sphygmomanometrically and cardiovascular risk factors making up the criteria for the metabolic syndrome were assessed.

**Main outcome measures:**

Systolic (SBP) and diastolic (DBP) blood pressure reduction, – response, and – normalization (systolic and diastolic), changes in fasting glucose, waist circumference (abdominal obesity), serum triglycerides and HDL cholesterol as well as the proportion of patients fulfilling the criteria for the metabolic syndrome. Number and nature of adverse events (AEs).

**Results:**

After 9 month the use of *Irbesartan in monotherapy *resulted in a significant reduction of blood pressure (SBP: -26.3 ± 10.1 mmHg/DBP-13.0 ± 6.6 mmHg, both p < 0.0001) in patients with the metabolic syndrome. This was accompanied by a reduction in cardiovascular risk factors: HDL cholesterol (+3.6 ± 7.2 mg/dl in men, +3.8 ± 6.5 mg/dl in women, both p < 0.0001), serum triglycerides (-28.6 ± 52.1 mg/dl, p < 0.0001), fasting blood glucose (-8.4 ± 25.1 mg/dl, p < 0.0001) and waist circumference (-2.4 ± 11.9 cm in men, -1.2 ± 14.2 in women, both p < 0.0001) were significantly improved. *Irbesartan combination therapy (12.5 mg HCTZ) *in patients with the metabolic syndrome: blood pressure reduction (SBP: -27.5 ± 10.1 mmHg/DBP: -14.1 ± 6.6 mmHg, both p < 0.0001), improvement in HDL cholesterol (+4.0 ± 6.8 mg/dl in men, +3.4 ± 6.8 in women, both p < 0.0001), triglycerides (-34.1 ± 52.6 mg/dl, p < 0.0001), fasting blood glucose (-10.0 ± 24.7, p < 0.0001) and waist circumference (-3.2 ± 12.7 cm in men, -1.7 ± 14.4 in women, both p < 0.0001). *Tolerability *was excellent: only 0.6% of patients experienced an AE.

**Conclusion:**

There was a significant improvement in blood pressure and metabolic risk factors as a result of Irbesartan treatment. There was no evidence of a difference between monotherapy and combination therapy with regard to the cardiovascular risk profile.

## Background

"Metabolic syndrome" describes the presence of a cluster of cardiovascular risk factors including hypertension, insulin resistance or glucose intolerance, visceral obesity and atherogenic dyslipidemia, resulting in a prothrombotic and proinflammatory state [1,2]. The presence of the metabolic syndrome predicts a two- to four-fold increase in the risk of cardiovascular disease and death [3,4], and the risk of developing type 2 diabetes is increased five- to nine-fold [5,6].

The lack of a universally agreed definition has complicated the epidemiologic research on the prevalence of this syndrome [7]. Nevertheless, it has been proposed that the metabolic syndrome is present in about 10–25% of individuals in the industrialized world [5,8]. The availability of high-calorie, low-fiber foods and more sedentary lifestyles are also leading to an increase in the prevalence of the metabolic syndrome in developing countries [9]. Recent data from the German Metabolic and Cardiovascular Risk Study (GEMCAS) [10] indicated a prevalence of 28% for the German primary care population (34% in men, 24% in women) using the AHA, NHLBI Definition 2005 [7] – a population that is also investigated in the current study.

In general, risk factors of the metabolic syndrome are treated separately and there is currently no available treatment that targets all components. Some classes of antihypertensive drugs, notably calcium channel blockers, angiotensin converting enzyme (ACE) inhibitors and angiotensin II receptor blockers (ARBs), have been shown to reduce or at least not to increase the incidence of new-onset diabetes, particularly as compared to diuretics and betablockers [11]. This suggests that antihypertensive agents may have differential effects on hyperglycemia in patients with metabolic syndrome. Furthermore, recent work has shown that Irbesartan and Telmisartan act as partial peroxisome proliferator-activated receptor gamma (PPARγ) agonists at concentrations that are achievable with oral doses recommended for the treatment of hypertension, thus suggesting their insulin-sensitizing effect [12-14]. Comparing the two ARBs Telmisartan and Losartan in a clinical study, Vitale et al. were recently able to show that Telmisartan, unlike Losartan, was able to reduce free plasma glucose, free plasma insulin, and HbA1c, suggesting a general intra class difference in the potential for improving the metabolic abnormalities present in patients with the metabolic syndrome [15].

It was therefore the aim of the present analysis of the post authorization study *Treat to Target *to investigate in more detail the influence of the PPARγ activating ARB Irbesartan with or without HCTZ on metabolic parameters. It was conducted as an observational study in primary care in order to acquire a broad spectrum of patients in clinical practice. The intention was to investigate three core questions: 1) Characteristics and comorbidity pattern of patients with the metabolic syndrome, 2) Blood pressure response to Irbesartan alone and in combination with hydrochlorothiazide (including response, overall and systolic/diastolic normalization), and 3) accompanying changes in cardiovascular risk factors (components of the metabolic syndrome) in monotherapy vs. combination therapy.

## Methods

### Design

This was a 9-month, multicenter, open-label, two-armed, prospective, observational post-authorization survey (PAS), which was conducted by 3,609 general physicians, practitioners and internists (GPs) throughout Germany. This specific study type is regulated by the German Drug Law (AMG) §67(6) and is primarily intended to gather knowledge about the safety and efficacy of marketed drugs in daily practice. The federal panel doctors' associations as well as the higher authorities were duly notified about this investigation. The participating GPs received a small remuneration for the documentation of each patient, which is common practice for this type of PAS. Importantly, as in any PAS, the protocol stipulated no interventions different from routine treatment. In a PAS the drug can only be prescribed in the labeled indication according to the Summary of Product Characteristics (SmPC). The procedures and decisions of the physicians were not influenced and the physicians were completely free to select which patients to treat with the licensed drug being studied, which diagnostic measurements they used, and the way in which they monitored the course of treatment or which concurrent or additional medication they prescribed. Due to the non-interventional type of the study, no ethics committee approval or patient informed consent has to be obtained in accordance with the German local laws and regulations. The participating physicians collected data on the background characteristics of the patients and on key efficacy variables and adverse events (AEs) and documented them in case report forms (CRFs). If any serious adverse events (SAEs) occurred, the GPs were obliged to report them by completing a form within 24 hours and transmitting it to the manufacturer, who then forwarded the report in a standardized format to the relevant authority (Bundesinstitut für Arzneimittel und Medizinprodukte, Bonn, Germany). The collected data, SAE forms and CRFs were not consistently verified in comparison with the source data in the patient files, but the forms were systematically screened for plausibility and completeness.

### Patients and Study Conduct

Patients with an indication for treatment with Irbesartan with or without HCTZ were selected by the GPs, using a cohort approach. Only adult patients could be included (18 years), and there were no additional exclusion criteria regarding concomitant medication or concomitant diseases except those specified in the SmPC. Patients were stratified into the metabolic syndrome cohort if they fulfilled the criteria set forth by the National Cholesterol Education Program Expert Panel (NCEP) in 2001 [2], which applied to 9281 patients. 4919 patients with uncontrolled arterial hypertension without the metabolic syndrome served as controls (the intention was to have a 2 : 1 distribution). The GPs selected patients with uncontrolled arterial hypertension for once daily treatment with Irbesartan (Aprovel™ 75, 150 or 300 mg) as monotherapy or in a combination with 12.5 mg hydrochlorothiazide (HCTZ) (CoAprovel™ 150/12.5 or 300/12.5 Sanofi-Aventis Deutschland GmbH, Berlin). The prescription of additional antihypertensive agents was allowed, as was the discontinuation of other medications, if necessary. There were no stipulations regarding BP targets. However a considerable percentage of German doctors follow the established guidelines of the German Hypertension League, which are < 140/90 mmHg for all patients except patients with diabetes, for whom < 130/80 mmHg applies [16].

The parameters documented in the study included demographic characteristics (initials, age, sex, weight and height, hip and waist circumference, familial cardiovascular disease and smoking status), medical diagnoses (diabetes, arterial hypertension with the number of years present and known microalbuminuria/proteinuria). Blood pressure was to be taken as a mean of three sphygmomanometric measurements, and pulse rate (beats per minute, bpm) and the respective target blood pressure for each patient were also to be recorded. If available from the charts, the following laboratory parameters were collected: fasting blood glucose, HbA1c, triglycerides, HDL and LDL cholesterol, creatinine and urinary albumin. In addition, concomitant disease was documented: stroke/transient ischemic attack (TIA), neuropathy, coronary heart disease (CHD), heart failure, previous myocardial infarction (MI), aorto-coronary venous bypass operation (ACVB), retinopathy, previous PTCA/stent, left ventricular hypertrophy (LVH), lipid disorders and peripheral arterial disease (PAD). Antihypertensive therapy within the previous 12 months was documented as well as the modifications after switching medications at the baseline visit.

After 3 and 9 months blood pressure measurements were repeated and the following parameters obtained if available: weight, hip and waist circumference, pulse, triglycerides, HDL and LDL cholesterol, fasting blood glucose, HbA1c, creatinine and urinary albumin. Modifications of antihypertensive therapy were documented and whether patients reached blood pressure targets was determined.

The following features of AEs were recorded if these occurred: description, first occurrence, grade of severity, outcome of events (recovered, recovered with sequelae, unresolved), likelihood of causal relationship (possible, probable, improbable, no relationship).

### Statistical Analyses

According to the predefined statistical analysis plan, the statistical analysis was performed descriptively and was interpreted in an explorative way. Comparisons were made for blood pressure and proportions of patients with components of the metabolic syndrome positive between baseline and the two post-baseline visits. The absolute and relative frequencies of AEs and the efficacy and tolerability ratings were reported. Post-hoc analyses for subgroups defined by gender, BMI, waist circumference, duration of hypertension, strength of antihypertensive response, and previous and concomitant antihypertensive treatment, respectively, were carried out. The analysis of data was performed with the statistical software package SAS, version 8.2. Test applied are indicated in the legend of tables and figures [17]. Regarding safety, the trial was adequately sized to identify rare AEs, i.e. those that may not have been detected in previous clinical studies, (incidence 1: 1,000) with a probability of > 99% and very rare events (incidence 1: 10,000) with a probability of > 75%.

## Results

### Baseline Characteristics

In the observational period between January 2005 and July 2006, a total of 14,200 patients were documented, of which 9281 were diagnosed as having the metabolic syndrome (MS); 4919 patients served as controls. Men and women were balanced (52.4 vs. 46.3% in patients without the MS, 51.8 vs. 47.9% in patients with the MS, with a mean age of 61.2 ± 11.6 (without MS) and 62.4 ± 10.2 years (with MS). Mean BMI was 26.8 ± 3.7 kg/m^2 ^without and 31.2 ± 5.0 kg/m^2 ^with the MS. Accordingly, both male and female patients with the MS had a higher waist circumference: men 111.3 ± 12.8 vs. 96.9 ± 10.2 cm and women 100.7 ± 14.6 vs. 85.9 ± 11.9 cm.

**Figure 1:**
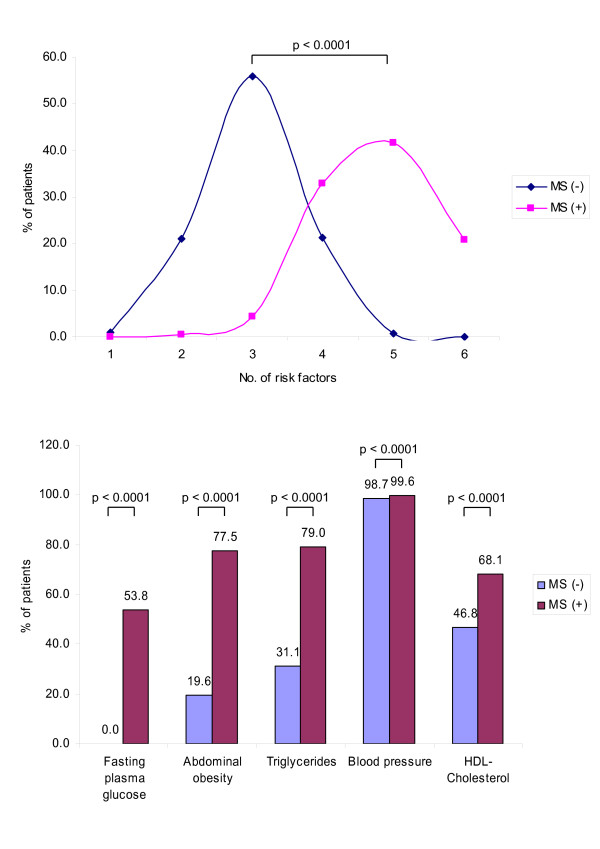
**a&b: **Risk factor distribution (Figure 1a) and presence of metabolic risk factors in patients with or without the metabolic syndrome (Figure 1b). Fasting plasma glucose 110 mg/dL; Abdominal obesity > 102 cm in men/> 88 cm in women; Triglycerides ≥ 150 mg/dL; Blood Pressure ≥ 130/≥ 85 mmHg; HDL cholesterol: < 40 mg/dL in men, < 50 mg/dL in women; Met (+) – Patients with the metabolic syndrome (doctors diagnosis); Met (-) – Patients without the metabolic syndrome; statistical test applied: Chi^2^-test

Figure 1 shows the risk factor pattern of patients with the MS in comparison to patients without. While an elevated blood pressure was an inclusion criterion for this observational study (e.g. ~ 100% default) all other risk factors are substantially increased in patients with the metabolic syndrome – abdominal obesity (77.5 vs. 19.6%, p < 0.0001) and fasting glucose (53.8 vs. 0.0%, p < 0.0001) being the most apparent difference between the two groups.

### Comorbidity pattern of patients with or without the metabolic syndrome

**Figure 2:**
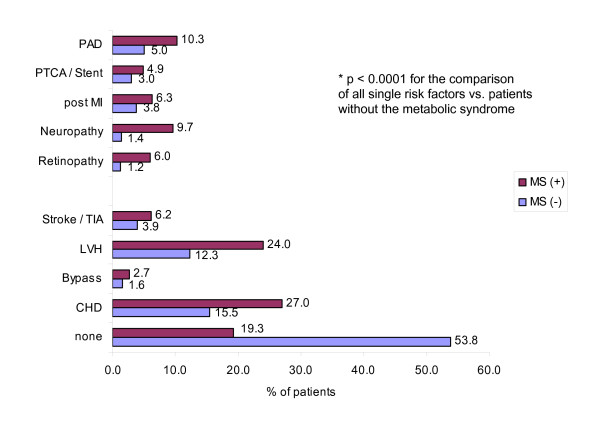
Comorbidity pattern of patients with or without the metabolic syndrome (Total n = 14,200). PAD – peripheral arterial disease; PTCA – Percutaneous transluminal coronary angiography; MI – Myocardial infarction; TIA – Transient ischemic attack; LVH – Left ventricular hypertrophy, CHD – Coronary heart disease; Met (+) – Patients with the metabolic syndrome (doctors diagnosis); Met (-) – Patients without the metabolic syndrome; statistical test applied: Chi^2^-test

In line with the documentation of cardiovascular risk factors is the observation that patients that have been assigned the diagnosis metabolic syndrome by their treating physicians have many more comorbid conditions than their controls (see figure 2). There are far fewer patients with no comorbidities (19.3% vs. 53.8%, p < 0.0001). In general, the prevalence of the comorbidities listed is at least twice as high as in control patients, retinopathy being increased five-fold (6.0 vs. 1.2%, p < 0.0001) and neuropathy about seven-fold (9.7 vs. 1.4%, p < 0.0001).

### Antihypertensive medication

At baseline, patients with the metabolic syndrome were receiving more medication to lower blood pressure as compared to their corresponding controls (see table 1). In particular, ACE-inhibitors (+ 11.3%), diuretics (+17.1%) and calcium antagonists (+8.9%) were being prescribed more frequently at the time of the baseline visit (before any change in medication, p < 0.0001). When switching to the ARB medication, in most cases previous ACE inhibitor use is discontinued (59.3 down to 7.7% in patients with the MS) but also diuretics are withdrawn in many cases. In line with this, the most frequently used new therapy is Irbesartan 300 mg/12.5 mg HCTZ, which is instituted in more than 60% of cases with the MS. In general, approximately 80% of all patients receive 300 mg Irbesartan – either alone or in combination with HCTZ.

**Table 1:** Antihypertensive therapy at baseline and during follow-up

		**Baseline**		**after switch**		**after 3 month**		**after 9 month**	
		**Metab (-)**	**Metab (+)**	**Metab (-)**	**Metab (+)**	**Metab (-)**	**Metab (+)**	**Metab (-)**	**Metab (+)**

**Substance classes**									

none	%	4.9	2.7	0.0	0.0	0.0	0.0	0.0	0.0
									
ACE-inhibitors	%	48.2	59.3*	3.7	7.7*	3.5	7.6*	3.5	7.0*
Alpha-blockers	%	3.2	4.1**	1.6	2.6*	1.5	2.6*	1.5	2.6*
ARBs	%	4.0	4.7	98.9	99.8*	97.6	97.7	91.9	92.0
Beta-blockers	%	44.3	49.3*	25.9	34.8*	25.3	33.7*	24.2	31.3*
Diuretics	%	30.7	47.8*	7.0	14.4*	6.5	13.6*	6.0	12.4*
Calcium antagonists	%	27.5	36.4*	13.3	22.7*	13.4	23.0*	13.0	21.8*
									
Irbesartan 75 mg	%	< 0.1	0.0	0.3	0.2	0.3	0.1**	0.3	0.1**
Irbesartan 150 mg	%	0.5	0.4	11.0	5.8*	8.0	3.7*	7.5	3.2*
Irbesartan 300 mg	%	0.1	0.2	29.8	22.0*	28.2	19.8*	25.2	17.9*
Irbesartan 150 mg/12,5 mg HCTZ	%	< 0.1	0.1	10.8	9.1**	8.9	6.6*	8.4	6.1*
Irbesartan 300 mg/12,5 mg HCTZ	%	0.1	< 0.1	46.0	62.1*	50.4	66.0*	48.2	62.6*

Met (+) – Patients with the metabolic syndrome (doctors' diagnosis); Met (-) – Patients without the metabolic syndrome; statistical test applied: Chi^2^-test; * p < 0.0001 vs. patients without MS; ** p < 0.01 vs. patients without MS

### Metabolic risk factors – Effects in patients treated with Irbesartan with or without the metabolic syndrome

Comparing the effect of Irbesartan (alone or in combination with HCTZ) on metabolic risk factors in patients with vs. in patients without the metabolic syndrome, remarkable differences can be found. While there is no effect of treatment on HDL cholesterol in women without the metabolic syndrome (-0.3 ± 5.8 mg/dL, p = ns), in men there is an increase of 0.8 ± 6.2 mg/dL (p < 0.0001). In patients with the metabolic syndrome there is a significant increase in both genders (+3.3 ± 6.7 mg/dL in women, p < 0.0001 and +3.8 ± 7.0 mg/dL in men, p < 0.0001). Likewise there is no clinical benefit on fasting plasma glucose in normal patients (+0.3 ± 13.5 mg/dL, p = ns after 3 months; +1.1 ± 15.0 mg/dL, p < 0.05 after 9 months) as compared to metabolic syndrome patients (-9.0 ± 25.0 mg/dL, p < 0.0001 after 9 months). All other comparisons are significant and show an improvement in the parameters documented, but still, patients with the metabolic syndrome seem to benefit more from Irbesartan treatment than patients without (see table 2).

**Table 2:** Metabolic risk factors in patients treated with Irbesartan with or without the metabolic syndrome

		**Patients without the metabolic syndrome**^1^							**Patients with the metabolic syndrome**^1^						
Components of the Met. Syn.		Baseline		3 months		9 months		Reduction †	Baseline		3 months		9 months		Reduction †

Blood pressure		mean	± SD	mean	± SD	mean	± SD		mean	± SD	mean	± SD	mean	± SD	

															
systolic	mmHg	159.4	± 13.3	136.8*	± 11.3	131.9*	± 10.0	-27.5	160.7	± 13.4	139.1*	± 11.5	133.9*	± 10.5	-26.8
diastolic	mmHg	93.7	± 8.5	82.6*	± 7.5	79.9*	± 6.7	-13.8	94.3	± 8.8	83.8*	± 7.6	80.8*	± 6.8	-13.5
															
Fasting plasma															
glucose	mg/dL	91.5	± 13.6	91.8	± 13.5	92.6**	± 15.0	1.1	120.9	± 30.1	114.2*	± 26.3	111.9*	± 25.0	-9.0
															
Abdominal obesity															
men	cm	96.6	± 10.2	96.1*	± 10.2	95.9*	± 10.1	-0.7	111.3	± 12.8	109.9*	± 12.4	108.6*	± 12.6	-2.7
women	cm	85.9	± 11.9	85.5*	± 11.7	85.8**	± 11.8	-0.1	100.7	± 14.6	100*	± 14.6	99.2*	± 14.3	-1.5
															
Triglycerides	mg/dL	153.3	± 42.2	151.9*	± 40.2	151.7*	± 41.1	-1.6	217.5	± 62.5	196.6*	± 56.2	186.7*	± 53.2	-30.8
															
HDL cholesterol															
men	mg/dL	48.6	± 6.2	49.2*	± 6.1	49.4*	± 6.2	0.8	41.8	± 7.6	44.2*	± 7.1	45.6*	± 7.0	3.8
women	mg/dL	52.2	± 5.6	52.2	± 5.8	51.9	± 5.8	-0.3	44.4	± 7.4	46.6*	± 6.9	47.7*	± 6.7	3.3

^1 ^doctors' diagnosis; statistical test applied: t-test; * p < 0.0001; ** p < 0.05; † reduction versus baseline

### Components of the metabolic syndrome – Irbesartan alone or in combination with HCTZ

To assess the suitability of Irbesartan combination therapy with HCTZ for use in patients with the metabolic syndrome the same parameters as above were assessed and patients on monotherapy were compared with those on the combination. There were consistent and highly significant (p < 0.0001) reductions of blood pressure, fasting plasma glucose, abdominal obesity, triglycerides and an increase in HDL cholesterol after 9 months of treatment with Irbesartan monotherapy as well as with the combination therapy (for individual figures see table 3). While individual figures differed slightly between groups there was no consistent trend to signal untoward effects of the combination therapy with HCTZ.

**Table 3:** Metabolic risk factors in metabolic syndrome patients with Irbesartan treatment alone or in combination with HCTZ

		**Irbesartan in patients with the metabolic syndrome**^1^							**Irbesartan/12.5 mg HCT in patients with the metabolic syndrome**^1^						
**Components of the Met. Syn.**		Baseline		3 months		9 months		Reduction †	Baseline		3 months		9 months		Reduction †

Blood pressure		mean	± SD	mean	± SD	mean	± SD		mean	± SD	mean	± SD	mean	± SD	

															
systolic	mmHg	159.1	± 12.9	137.8*	± 11.2	132.8*	± 10.1	-26.3	161.3	± 13.2	139.0*	± 11.2	133.8*	± 10.1	-27.5
diastolic	mmHg	93.4	± 8.6	82.8*	± 7.2	80.4*	± 6.6	-13.0	95.0	± 8.6	83.8*	± 7.4	80.9*	± 6.6	-14.1
															
Fasting plasma															
glucose	mg/dL	118.3	± 29.9	111.8*	± 26.0	109.9*	± 25.1	-8.4	122.9	± 29.8	115.4*	± 26.0	112.9*	± 24.7	-10.0
															
Abdominal obesity															
men	cm	109.9	± 13.0	108.4*	± 12.2	107.5*	± 11.9	-2.4	112.0	± 12.9	110.3*	± 12.3	108.8*	± 12.7	-3.2
women	cm	99.3	± 14.3	98.3*	± 14.3	98.1*	± 14.2	-1.2	101.5	± 14.7	100.8*	± 14.7	99.8*	± 14.4	-1.7
															
Triglycerides	mg/dL	212.3	± 61.2	194.2*	± 56.7	183.7*	± 52.1	-28.6	221.5	± 63.0	198.4*	± 55.3	187.4*	± 52.6	-34.1
															
HDL cholesterol															
men	mg/dL	42.2	± 7.7	44.6*	± 7.3	45.8*	± 7.2	3.6	41.4	± 7.5	44.0*	± 7.0	45.4*	± 6.8	4.0
women	mg/dL	45.0	± 7.2	47.8*	± 6.8	48.8*	± 6.5	3.8	44.2	± 7.4	46.4*	± 6.9	47.6*	± 6.8	3.4

^1 ^doctors' diagnosis; statistical test applied: t-test; * p < 0.0001; ** p < 0.05; † reduction versus baseline

**Figure 3:**
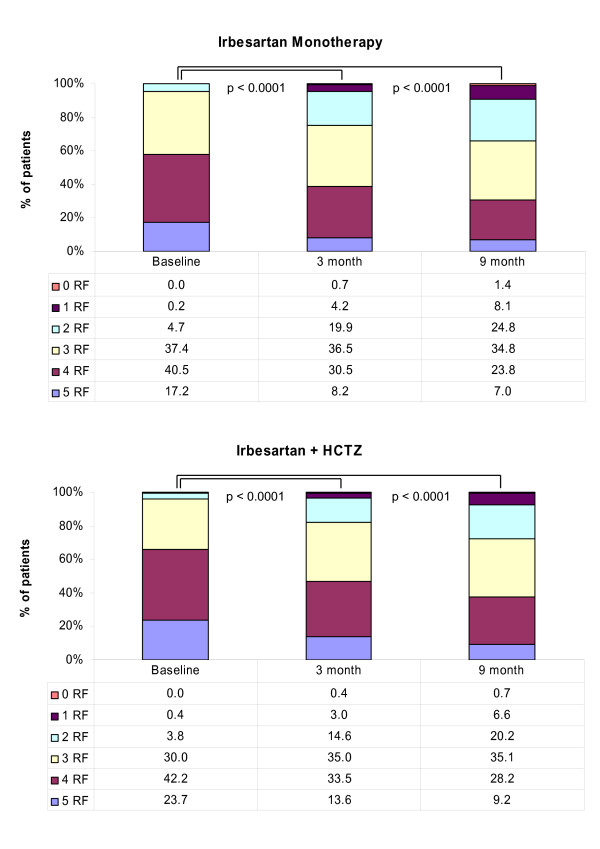
**a&b: **Number of metabolic risk factors present in metabolic syndrome patients with Irbesartan alone (Figure 3a) or in combination with HCTZ (Figure 3b). p < 0.0001 for the comparison of 3-month and 9-month to baseline; statistical test applied: Chi^2^-test

Consequently there was a marked reduction in the number of patients fulfilling the criteria for the metabolic syndrome (-29.5% for patients on monotherapy, -23.4% for patients on fixed combinations with HCTZ) irrespective of whether hypertension was considered or not. Figure 3 shows that while almost all patients were considered to have the metabolic syndrome (based on baseline risk factor prevalence (95.1% in the group with monotherapy, 95.8% in the combination group), the proportion of patients not fulfilling the criteria for the metabolic syndrome rose from 4.9% and 4.2% respectively to 34.3% (monotherapy) and 27.5% (combination).

### Side effects

The rate of AEs was very low. Only 141 AEs were noted in 88 patients (0.62% of all patients). 65 SAEs were noted in 34 patients (0.24%). There were 17 deaths in 14,200 patients during a study period of 9 months. For details see Table 4.

**Table 4:** Most frequent serious side effects noted during the study (n)

Reported Side Effects	Irbesartan	Irbesartan/HCTZ
1 Cardiogenic shock	1	2
2 Cerebral infarction	1	1
3 Gastrointestinal hemorrhage	1	1
4 Metastatic bronchial carcinoma	1	1
5 Myocardial infarction	2	1
6 Rash	1	1
7 Tachyarrhythmia	1	1
8 Vertigo	1	1

out of a total of 14,200 patients

## Discussion

In the present study 14,200 unselected primary care patients with or without the metabolic syndrome were treated with Irbesartan alone or in combination with 12.5 mg HCTZ. The observation of the 9-month treatment period and the comparison of monotherapy and combination therapy in terms of blood pressure reduction and interference with cardiovascular risk factors yielded the following results: 1) There was a significant improvement in metabolic risk factors as a result of Irbesartan treatment. 2) There was no evidence of a difference between monotherapy and combination therapy with regard to the cardiovascular risk profile. 3) There was a pronounced blood pressure lowering effect with both monotherapy and combination therapy, with a slightly better blood pressure response in patients without the metabolic syndrome. 4) There was a favorable reduction in the number of tablets to be taken.

### Cardiovascular risk profile

During the 9-month study period an improvement in metabolic parameters was noted that was substantial for fasting plasma glucose, triglycerides and HDL cholesterol (patients with the metabolic syndrome > patients without). Interestingly, even a reduction in abdominal obesity was noted; this was small, but resulted in a reduction in waist circumference of up to 3.2 cm (in patients on Irbesartan/HCTZ combination therapy after 9 months).

It cannot be ruled out that the observed effects are due to the study conduct that may have improved patient compliance, but several lines of reasoning point toward a metabolic profile of Irbesartan that may be in part responsible for these results. 1) Both Telmisartan and Irbesartan activate the PPARγ receptor [12,14] and the EC50 value for transactivation is about 27 μmol/l for Irbesartan [14]. Clinical evidence for an insulin sensitizing effect of Irbesartan is lacking; however, administration of Irbesartan to insulin resistant genetically obese Zucker rats significantly decreased serum insulin levels and increased serum adiponectin levels compared to control animals treated with the vehicle. Fasting glucose levels were unaffected in this model [18,19]. 2) Since PPARγ not only improves insulin sensitivity but also improves the lipid metabolism, the observed effects on the lipid profile may also be related to PPARγ activation [20,21]. An increase in adiponectin levels has also been observed with ARBs [18,22]; they have been reported to be linked to an elevation in HDL cholesterol, an observation that supports the present finding [23]. On the other hand, in diabetic Zucker rats there was no effect on triglycerides or body weight [19]. 3) Irbesartan and other RAS blocking agents are more favorable in terms of body weight development than beta-blockers or diuretics. Even among the ARBs there may be a difference in body weight development as recently suggested by Sugimoto and colleagues [24]. In line with this reasoning a moderate reduction in weight loss cannot be ruled out and Irbesartan may be particularly worthwhile for antihypertensive treatment in overweight patients.

Another interesting observation is that the thiazide component when given in low dose together with Irbesartan did not lead to unfavorable consequences in terms of metabolic control. Whereas thiazide diuretics have proved to be highly effective blood pressure agents [25], HCTZ is known to increase insulin resistance and, in certain settings, can lead to adverse metabolic changes as well as increased plasminogen activator inhibitor-1, at least in higher doses [26,27]. Consequently, its use has been discouraged in susceptible populations such as patients with diabetes [28]. In contrast, ARBs have been shown to have beneficial effects on multiple components of the metabolic syndrome [29], an observation consistent with primary links between angiotensin II and insulin resistance [30]. Similarly HCTZ has been reported to abolish the antiatherosclerotic effects exerted by inhibition of the renin-angiotensin system with quinapril, at least in hypercholesterolemic rabbit models [31]. Given the high proportion of patients difficult to control with monotherapy and the value of diuretics in a variety of patient populations, it is of particular importance to counterbalance the untoward effects by using low dose combination therapy with an ARB. Furthermore, there is clear evidence from several endpoint trials including the LIFE study, that a combination of thiazide diuretics with ARB results in an effective reduction of cardiovascular risk and mortality in hypertensive subjects [32].

In conclusion, there seems to be a quantitative difference in the beneficial metabolic profile of Irbesartan between patients with and without the metabolic syndrome. Particularly patients with the metabolic syndrome benefit from the addition of Irbesartan to their antihypertensive regimen, an effect that is seen less so in normal patients but may even be less pronounced in diabetic patients, but this topic still awaits further studies.

### Blood pressure lowering effect

The observed blood pressure lowering effect with Irbesartan (for details see Table 2) is largely comparable to previous results obtained in an open label study in Switzerland in which 2621 previously treated or newly diagnosed patients achieved a mean blood pressure reduction of 25/13 mmHg after 4 months of treatment with Irbesartan (with or without HCTZ) [33]. It is slightly higher than in a German phase IV study in overweight and obese patients where blood pressure was lowered by 22/11 mmHg after 3 months of treatment [34], and in a recent open phase IV trial in the US where the combination of Irbesartan 300 mg with 25 mg HCTZ yielded a reduction of 21.5/10.4 mmHg; however, this latter was of shorter duration than the present study (18 weeks) [35]. The present study differed from these studies in that a much longer observation period was chosen, and between the 3-month documentation (which would correspond to the length of the studies cited above) and the 9-month there was an average additional blood pressure reduction of about 5 mmHg. Therefore the blood pressure lowering effect of Irbesartan with or without HCTZ can be regarded as clinically significant in the light of the need for aggressive blood pressure management in this patient population, where overall risk factor management is of foremost importance.

### Side effects

Irbesartan as monotherapy and in various combinations was well tolerated, as evidenced by the low rate of AEs (141 AEs in 88 patients, 0.62%). In previous open-label observational studies with ARBs in the primary-care setting, the respective rates had been considerably higher [36,37]. Interestingly, the rate did not differ significantly on comparison of the respective monotherapies or combination therapies with HCTZ.

### Limitations

The present results have to be considered against the background of potential limitations. The study was not controlled and therefore the contribution of placebo effects or the withdrawal of other antihypertensive agents is unknown. Second, in the absence of randomization procedures the influence of unknown biases, e.g. through patient selection, cannot be assessed. Third, concomitant medication influencing the metabolic profile (lipid lowering agents and oral antidiabetic agents or insulin) have not been documented in the present study, but since patients were mostly non-diabetic the latter two treatments are not likely to be prescribed in greater numbers. Among the strengths of the study was the choice of the setting. Observational studies in primary care, which include typical patient groups and reflect current treatment approaches, are useful for complementing the findings of randomized controlled trials [38].

## Conclusion

The present study demonstrates in a large patient cohort with or without the metabolic syndrome that treatment with an Irbesartan-based regimen for 9 months not only results in a pronounced blood pressure reduction, but also might have a favorable impact on important metabolic parameters such as HDL cholesterol, triglycerides and blood glucose. An Irbesartan-based regimen therefore seems to be a rational treatment for patients with the metabolic syndrome.
